# Infectious esophagitis in the immunosuppressed: Candida and beyond

**DOI:** 10.33582/2637-4900/1004

**Published:** 2018-04-05

**Authors:** Kais Zakharia, James H Tabibian

**Affiliations:** 1 Internal Medicine Residency Program, Department of Medical Education, Beaumont Health- Dearborn, Dearborn, MI, USA; 2 Division of Gastroenterology and Hepatology, Mayo Clinic, Rochester, MN, USA; 3 Division of Gastroenterology, Department of Medicine, Olive View-UCLA Medical Center, Sylmar, CA, USA

**Keywords:** Dysphagia, Odynophagia, Immunosuppression, Opportunistic infection, Endoscopy

## Abstract

Infection is the second most common cause of esophagitis, second only to gastroesophageal reflux, and represents a clinically important disorder. Immunosuppressed patients are at highest risk for infectious esophagitis, with *CANDIDA,* herpes simplex virus, and cytomegalovirus being the most common causative microorganisms. Here we provide a brief clinical review and present a case of concomitant oropharyngeal and presumed esophageal candidiasis in a patient with autoimmune hepatitis who was initiated on high-dose corticosteroid therapy and soon thereafter develop odynodysphagia and who was found to have herpes esophagitis diagnosed by endoscopy and histopathology.

## Background

Infectious esophagitis is the second most common cause of esophagitis, surpassed only by reflux esophagitis, and represents a clinically important and potentially serious condition. It occurs predominantly in immunocompromised patients (e.g. due to chemotherapy, corticosteroid use, HIV infection) or in the context of host microbiome alterations (e.g. antibiotic use) [1,2]. The most common causes of infectious esophagitis are *Candida,* herpes simplex virus (HSV), and cytomegalovirus (CMV). Candidal esophagitis typically occurs as an extension of oral candidiasis (i.e. thrush); while infection of only the oral cavity is frequently asymptomatic, extension into the esophagus generally results in dysphagia and/or odynophagia. HSV esophagitis occurs most commonly in transplant recipients. The vast majority of HSV esophagitis is due to HSV type 1, although type 2 HSV esophagitis has also been reported [1]. HSV esophagitis can result from the activation of the virus and spread through the vagal nerve or by direct spread from the oral mucosa into the esophageal mucosa [3]. CMV esophagitis is the most common cause of esophagitis in advanced HIV-infection (i.e. patients with acquired immune deficiency syndrome) [4].

In this report, we present the case of a patient receiving corticosteroid therapy for autoimmune hepatitis (AIH) who was found to have thrush and developed odynodysphagia thereafter but did not respond to appropriate antifungal treatment; further evaluation revealed acute herpetic esophagitis. The occurrence of simultaneous candidiasis and HSV esophagitis has not been previously reported, likely due to being under-recognized.

## Case report

**Figure 1:**
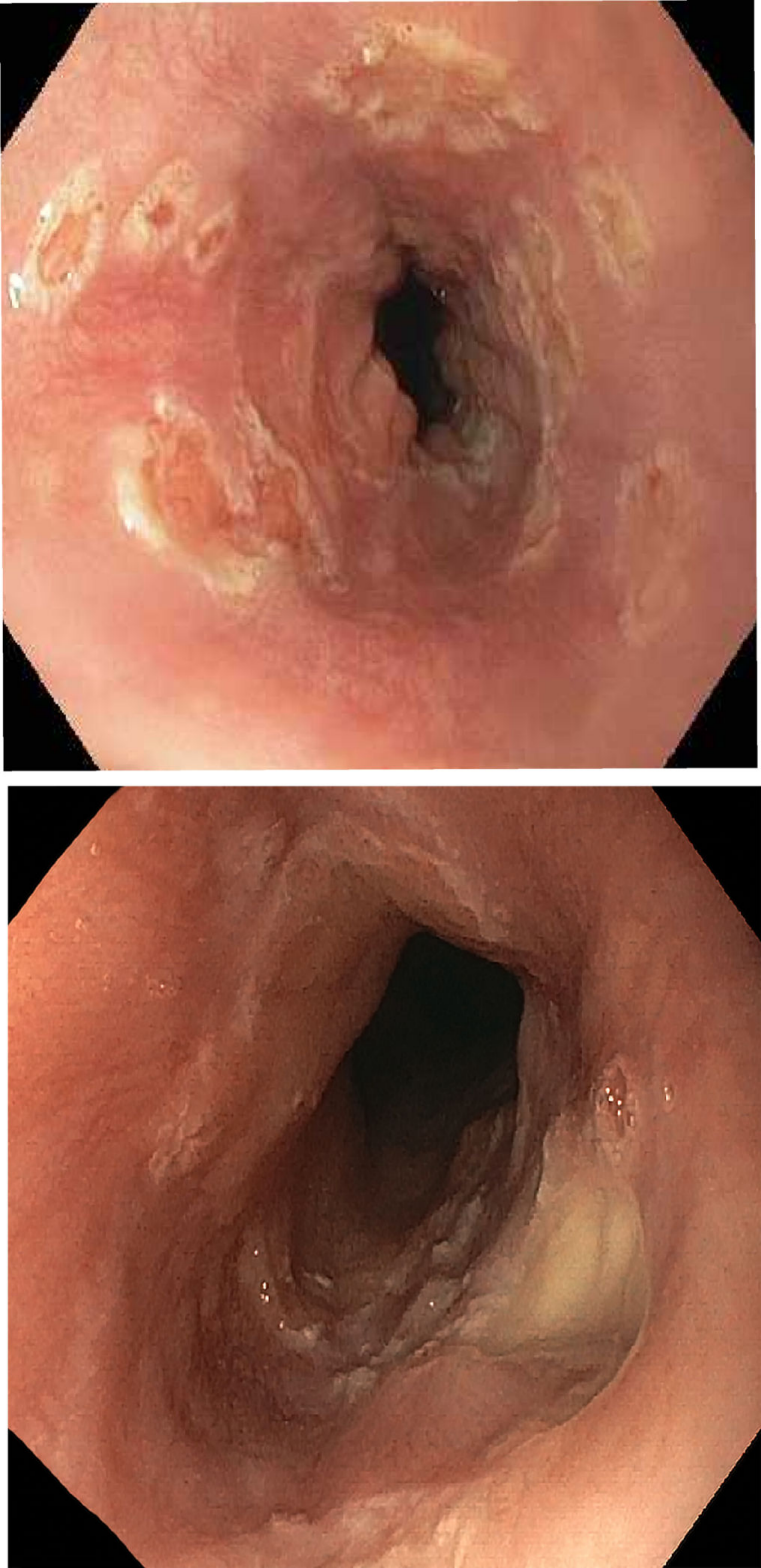
Endoscopic views of herpes esophagitis reveal diffuse mucosal erosions and ulcerations. a) Numerous subcentimeter punctate erosions and ulcerations throughout the esophagus. b) Clean-based circular ulceration in the distal esophagus.

**Figure 2:**
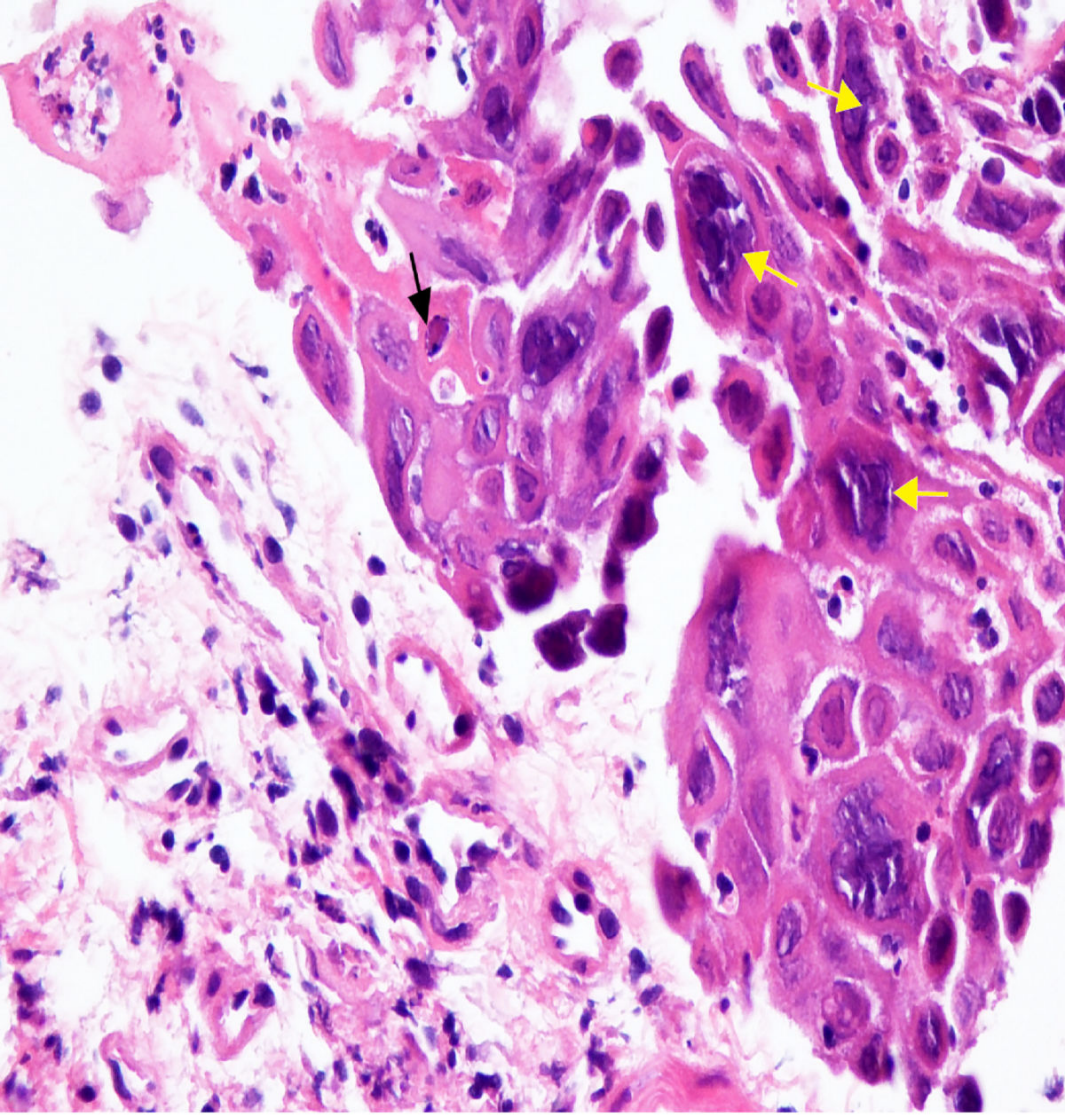
Histopathology of esophageal biopsies demonstrate multinucleated epithelial cells (yellow arrows) and Cowdry A inclusions (black arrow), indicative of herpesvirus infection.

A 58-year-old woman with cirrhosis secondary to AIH presented with progressive jaundice and darkening urine. Serum laboratory tests revealed total bilirubin 17.9 mg/dL, alkaline phosphatase 265 IU/mL, and alanine aminotransferase 613 IU/mL. Abdominal ultrasound showed changes of cirrhosis and portal hypertension. Oral prednisone 60 mg/day was prescribed for treatment of AIH flare. Ten days later, the patient developed sore throat and white oral plaques consistent with thrush; fluconazole therapy was initiated. Five days thereafter, the patient reported persistence of sore throat and new odynophagia. Physical examination revealed resolution of thrush but a new 5 mm gingival ulcer. Upper endoscopy was performed to assess for persistent of esophageal candidiasis and to rule out other potential etiologies of odynophagia; this demonstrated severe esophagitis with diffuse punctate ulcerations up to 1 cm in diameter (Figure 1a, b), from which cold forceps biopsies were obtained from both ulcer base as well as ulcer edge. Histopathology (Figure 2) demonstrated multinucleated epithelial cells (yellow arrows) and Cowdry A inclusions (black arrow), consistent with herpes virus infection. The patient was treated with acyclovir with rapid resolution of symptoms.

## Discussion

Although infectious esophagitis is a common clinical problem, to our knowledge, the present report represents the first well-substantiated case of concomitant oropharyngeal candidal infection and herpes esophagitis. The diagnosis of candidal esophagitis is often reliably predicted when a patient with odyno (dys) phagia is found to have thrush; indeed, most patients with thrush and odynophagia also have candidal esophagitis [5,6]. However, it is important to note that the absence of thrush cannot rule out the possibility of infectious esophagitis. Upper endoscopy with biopsy is the test of choice to confirm the diagnosis and rule out other possibilities, particularly in patients who do not respond to systemic antifungal therapy within a few days. Indeed, in a study of 72 HIV-positive infectious esophagitis patients; 20% had simultaneous *Candida* and CMV co-infection, 2% had candida, CMV, and HSV co-infection, and 2% had HSV and CMV co-infection; interestingly, none of the patients had co-infection of specifically *Candida* with HSV (i.e. without CMV, as appeared to be the case with our patient) [7].

On endoscopy, candidal esophagitis exhibits white or pale yellow mucosal plaque lesions; on the other hand, lesions in HSV esophagitis are ulcerated and have a cratered appearance (similar to CMV-related ulcers, though generally more numerous and not as large). On biopsy, candidal esophagitis shows yeasts and pseudohyphae invading the esophageal mucosa, whereas viral esophagitis is generally more subtle and may require a high index of suspicion coupled with special histopathological (including immunohistochemical) staining to confirm the presence of viral esophagitis and also distinguish between different viruses. This case, in addition to being a rare report of HSV and presumed candidal esophagitis within the same patient, highlights the essential clinical pearl for providers that candidal infection which does not exhibit symptom resolution within three days of treatment with a systemic antifungal agent warrants upper endoscopy with biopsy to confirm the diagnosis and rule out other co-infections such as HSV and CMV.

## Conclusion

This case serves as a reminder that persistent esophageal symptoms (e.g. odynophagia) following first-line antimicrobial therapy, particularly in immunocompromised hosts, merit further investigation with upper endoscopy.
